# Surgical resection could provide better outcomes for patients with hepatocellular carcinoma and tumor rupture

**DOI:** 10.1038/s41598-022-12350-x

**Published:** 2022-05-18

**Authors:** Chun-Yang Lee, Gar-Yang Chau, Cheng-Yi Wei, Yee Chao, Yi-Hsiang Huang, Teh-Ia Huo, Ming-Chih Hou, Yu-Hui Su, Jaw-Ching Wu, Chien-Wei Su

**Affiliations:** 1 Division of Gastroenterology and Hepatology, Department of Medicine, Taipei Veterans General Hospital, No. 201, Sec. 2, Shipai Rd., Peitou District, Taipei, 11217 Taiwan; 2 Department of Internal Medicine, School of Medicine, College of Medicine, National Yang Ming Chiao Tung University, Taipei, Taiwan; 3 Division of General Surgery, Department of Surgery, Taipei Veterans General Hospital, Taipei, Taiwan; 4 Department of Oncology, Taipei Veterans General Hospital, Taipei, Taiwan; 5 Institute of Clinical Medicine, College of Medicine, National Yang Ming Chiao Tung University, Taipei, Taiwan; 6 Division of Basic Research, Department of Medical Research, Taipei Veterans General Hospital, Taipei, Taiwan; 7 Institute of Pharmacology, College of Medicine, National Yang Ming Chiao Tung University, Taipei, Taiwan; 8 Department of Accounting, School of Business, Soochow University, Taipei, Taiwan; 9 Hospitalist Ward, Department of Medicine, Taipei Veterans General Hospital, Taipei, Taiwan; 10 Biomedical Science and Engineering Center, National Tsing Hua University, Hsinchu, Taiwan

**Keywords:** Gastroenterology, Hepatology

## Abstract

We investigated the outcomes of patients with ruptured hepatocellular carcinoma (HCC) and identified the optimal treatment modality for such patients. We retrospectively enrolled 91 patients with treatment-naive HCC and tumor rupture at diagnosis, including 38 patients who underwent surgical resection (SR) alone, 28 patients who were treated with transarterial chemoembolization (TACE) only, 20 patients who had a sequential combination therapy of TACE and SR, and 5 patients who received best supportive care. After a median follow-up of 13.1 months, 54 patients died. The cumulative 5 years overall survival (OS) rates were 55.1% and 0% in the SR group and non-SR group, respectively (*p* < 0.001). Non-SR therapy was associated with poorer OS according to a multivariate analysis with a hazard ratio of 6.649 (95% confidence interval 3.581–12.344, *p* < 0.001). Moreover, whether patients received TACE or not did not impact the OS in both the SR group and the non-SR group. In conclusion, for patients with HCC and tumor rupture at the time of diagnosis, SR could lead to better prognoses than non-surgery treatment modalities. Moreover, a sequential combination of TACE and SR had similar clinical outcomes when compared to SR alone.

## Introduction

Cancer is the second leading cause of death globally, and primary liver cancer now ranks the third most common cause of cancer mortality after lung cancer and stomach cancer^1^. It has been estimated that around 781,631 patients died from liver cancer in 2018^1^. Hepatocellular carcinoma (HCC) accounts for 90% of primary liver cancer and leads to approximately 700,000 deaths each year^2^. Moreover, spontaneous tumor rupture is the third most common cause of death among patients with HCC after tumor progression and liver failure^3^.

Rupture of HCC can be diagnosed by hemodynamic instability, hemoperitoneum on diagnostic paracentesis, or imaging studies, such as computed tomography (CT) scans or angiography^4,5^. Patients with ruptured HCC have significantly poorer prognoses than those who do not^6^. In the acute phase of HCC rupture, transarterial chemoembolization (TACE), surgical resection (SR), or a sequential combination therapy of TACE and SR are the major treatments for this life-threatening condition^6–8^. However, prognostic data on patients with ruptured HCC after different treatments are scarce. Thus, the aim of this study is to examine patients with ruptured HCC and identify the best treatment modality.

## Results

### Baseline demographic characteristics

**Figure 1:**
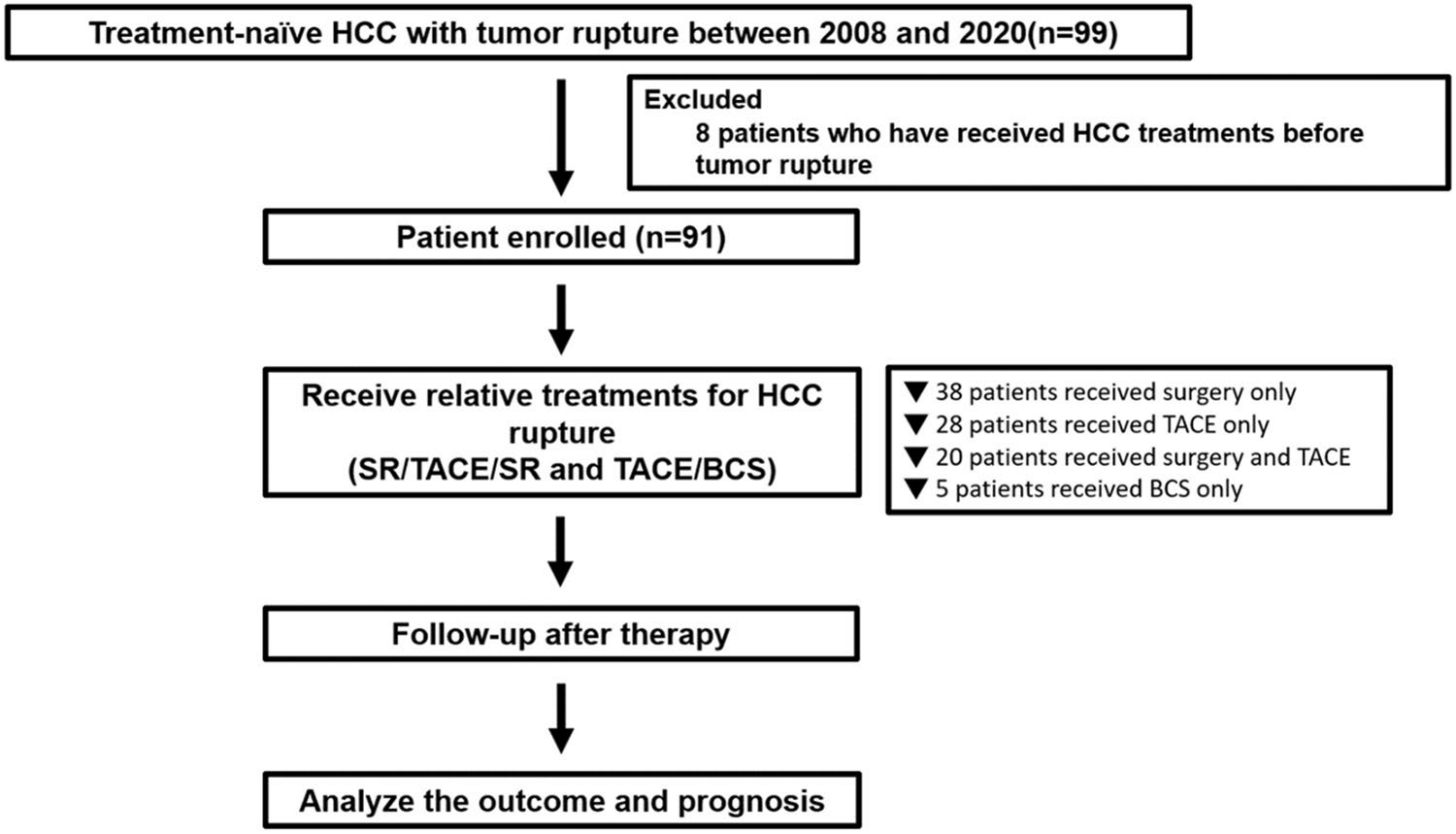
Study flow chart.

Among the 91 patients enrolled in this study (Fig. 1), 38 patients underwent SR only after the diagnosis of HCC rupture, 28 patients received TACE alone, 20 patients had a sequential combination therapy of TACE and SR, and the remaining 5 patients received best supportive care (BSC). We divided the patients into an SR group (including SR alone and TACE/SR combination therapy) and a non-SR group (including TACE alone and BSC). The median days between TACE and SR was 14 days (interquartile range (IQR) 6–49 days) among patients who underwent sequential combination therapy. Regarding the type of SR, 2 (3.4%) patients received wedge resection, 7 (12.1%) patients underwent sub-segmentary resection, 29 (50%) patients received segmentary resection, and the remaining 20 (34%) patients underwent lobectomy.

As shown in Table 1, compared to patients in the non-SR group, patients in the SR group had higher serum albumin levels, lower prothrombin-time international normalized ratio (PT INR), lower hemoglobin levels, lower blood urea nitrogen (BUN) levels, and lower serum creatinine levels. Moreover, they had higher rates of Child–Pugh class A and albumin-bilirubin (ALBI) grade 1. The other demographic characteristics, such as age, gender, viral etiology, and tumor size, were not statistically different between these two groups of patients. However, patients in the non-SR group had more advanced Barcelona Clinic Liver Cancer (BCLC) stage compared to those in the SR group.

**Table 1:** Baselines demographics of enrolled patients.

	All patients (n = 91)	SR (n = 58)	Non-SR (n = 33)	*p*
Age (years)	63.0, 50.0–76.0	62.0, 47.3–73.3	67.0, 51.0–79.5	0.110
Gender (M/F) (%)	71/20 (78.0/22.0)	45/13 (77.6/22.4)	26/7 (78.8/21.2)	1.000
AFP (ng/ml)	644.3, 14.8–28,436	673.8, 21.7–35,814	579.0, 11.5–16,419.6	0.245
Tumor size (cm)	8.6, 5.3–12.0	7.9, 5.3–10.2	10.0, 5.4– 13.5	0.063
HBsAg (+ /−) (%)	42/48 (46.7/53.3)	31/27 (53.4/46.6)	11/21 (34.4/65.6)	0.130
Anti-HCV (+ /−)(%)	16/73 (18.0/82.0)	8/49 (14.0/86.0)	8/24 (25.0/75.0)	0.315
Albumin(mg/dL)	3.60, 3.0–4.0	3.8, 3.3–4.1	3.0, 2.7–3.6	< 0.001
Bilirubin (U/L)	0.82, 0.60–1.48	0.76, 0.57–1.39	1.08, 0.61–1.72	0.066
Platelet (/mm^3^)	218,000, 136,000–267,000	222,000, 152,000–264,500	180,000, 101,000–272,500	0.102
PT INR	1.10 1.04–1.20	1.09, 1.02–1.14	1.16, 1.06–1.30	< 0.001
Hgb (mg/dL)	11.4, 9.1–13.2	11.9, 10.4–13.8	9.1, 8.1–11.3	< 0.001
BUN (mg/dL)	16.0, 12–23	15, 12.0–18.5	21.5, 12.8–31.0	0.003
Creatinine (mg/dL)	0.98, 0.81–1.28	0.95, 0.81–1.19	1.14, 0.80–11.3	0.017
ALT (U/L)	34.0, 24 − 71	30, 24 − 72	37, 27.5to − 91.5	0.306
ALBI	− 2.25, − 2.76 to − 1.69	− 2.53, − 2.79 to − 2.04	-1.68, − 2.24 to − 1.15	< 0.001
ALBI (1/2/3) (%)	33/43/15 (36.3/47.3/16.5)	27/28/3 (46.6/48.3/5.2)	6/15/12 (18.2/45.5/36.4)	< 0.001
Child–Pugh class (A/B/C) (%)	62/22/3 (71.3/25.3/3.4)	49/7/0 (87.5/12.5/0)	13/15/3 (41.9/48.4/9.7)	< 0.001
BCLC stage (A/B/C/D) (%)	11/44/29/7 (12.1/48.4/31.9/7.7)	10/35/13/0 (17.2/60.3/22.4/0)	1/9/16/7 (3.0/27.3/48.5/21.2)	< 0.001

Continuous variables are expressed as the median with 25th and 75th percentiles.

*SR* surgical resection, *TACE* trans-arterial chemoembolization, *BMI* body mass index, *AFP* α-fetoprotein, *HBsAg* hepatitis B surface antigen, *HCV* hepatitis C virus, *MELD* model for end-stage liver disease, *ALBI* albumin-bilirubin, *PT INR* prothrombin time/international normalized ratio, *HgB* hemoglobulin, *ALT* alanine aminotransferase, *GGT* γ-glutamyl transferase, *ALKP* alkaline phosphatase, *BCLC* Barcelona Clinic Liver Cancer.

The baseline demographic characteristics and BCLC stages were similar between patients who underwent SR alone and those with TACE and SR sequential combination therapy, except that patients in the SR group had higher hemoglobulin levels and more Child–Pugh class A liver functional reserve (Supplementary S1). Moreover, the demographic characteristics were comparable between patients who underwent TACE alone and those who received BSC (Supplementary S2).

### Factors related to OS

**Figure 2:**
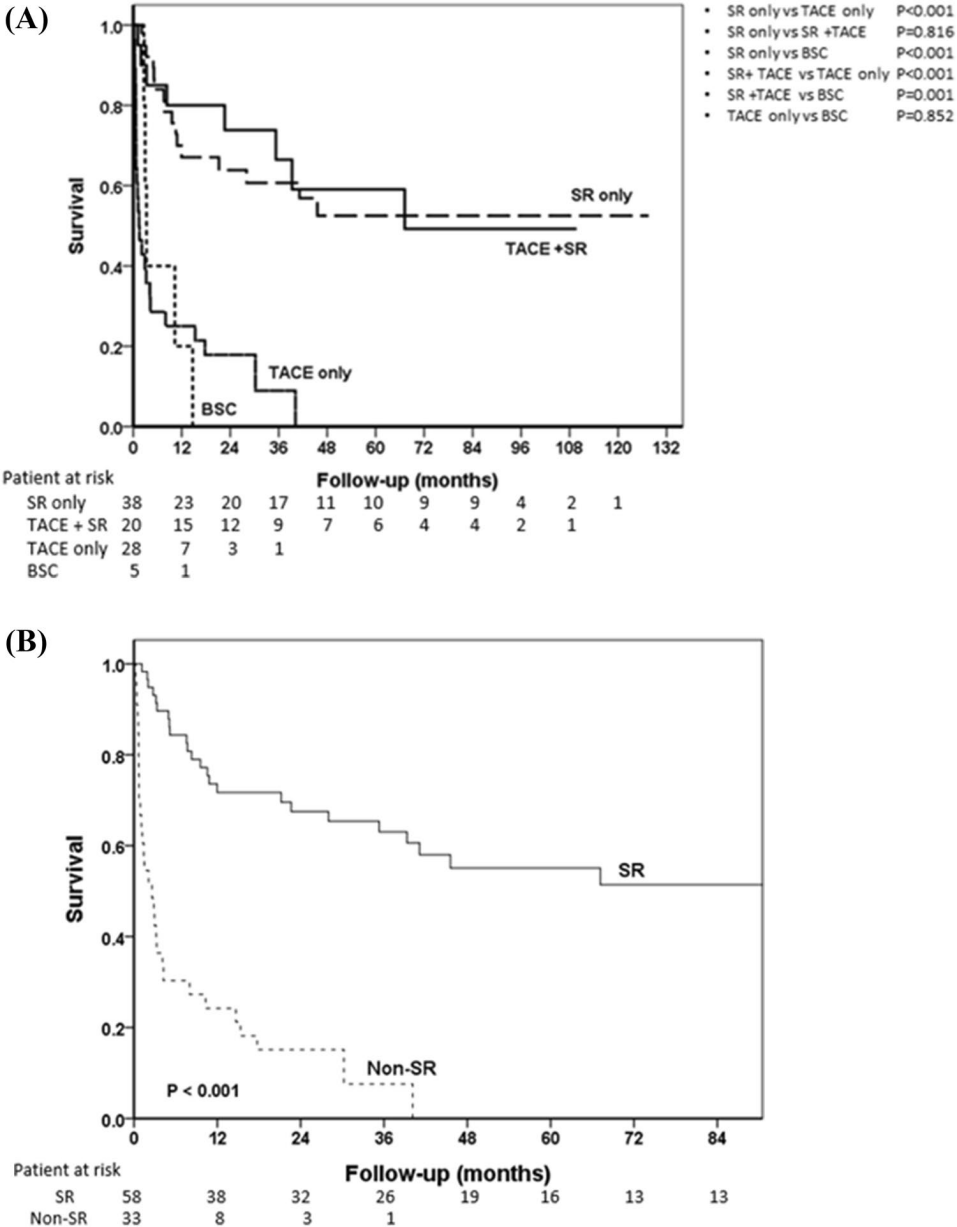
(**A**) Comparison of OS rates among HCC patients with different treatments, (**B)** Comparison of OS rates between SR and non-SR groups.

After a median follow-up period of 13.1 (IQR 2.9–41.1) months, 54 patients had died. The cumulative overall survival (OS) rates at 1 year, 2 years, 3 years, and 5 years were 54.5%, 48.0%, 43.6%, and 36.7%, respectively. Patients who underwent SR alone or a combination therapy of TACE and SR had significantly better OS than those who received TACE alone or BSC after HCC rupture **(**Fig. 2A). There were no significant differences in OS between patients who underwent SR alone and those who underwent TACE and SR sequential combination therapy (*p* = 0.816). The prognoses of patients were similar between TACE monotherapy and BSC (*p* = 0.852), which indicated that for patients with HCC rupture, surgical treatment is the major influence in the survival outcome.

As shown in Fig. 2B, the cumulative OS rates at 1 year, 2 years, 3 years, and 5 years for patients in the SR group and non-SR group were 71.7% versus 24.2%, 67.5% versus 15.2%, 63.0% versus 7.6%, and 55.1% versus 0%, respectively (*p* < 0.001). The median OS in the non-SR group was 2.6 months (95% confidence interval (CI) 0.9–4.3 months). Most of the patients in the non-SR group died within one year after a tumor rupture event, and no one survived for more than 4 years in the follow-up period.

### Comparison of OS between SR and non-SR group stratified by ALBI grade and BCLC stage

**Figure 3:**
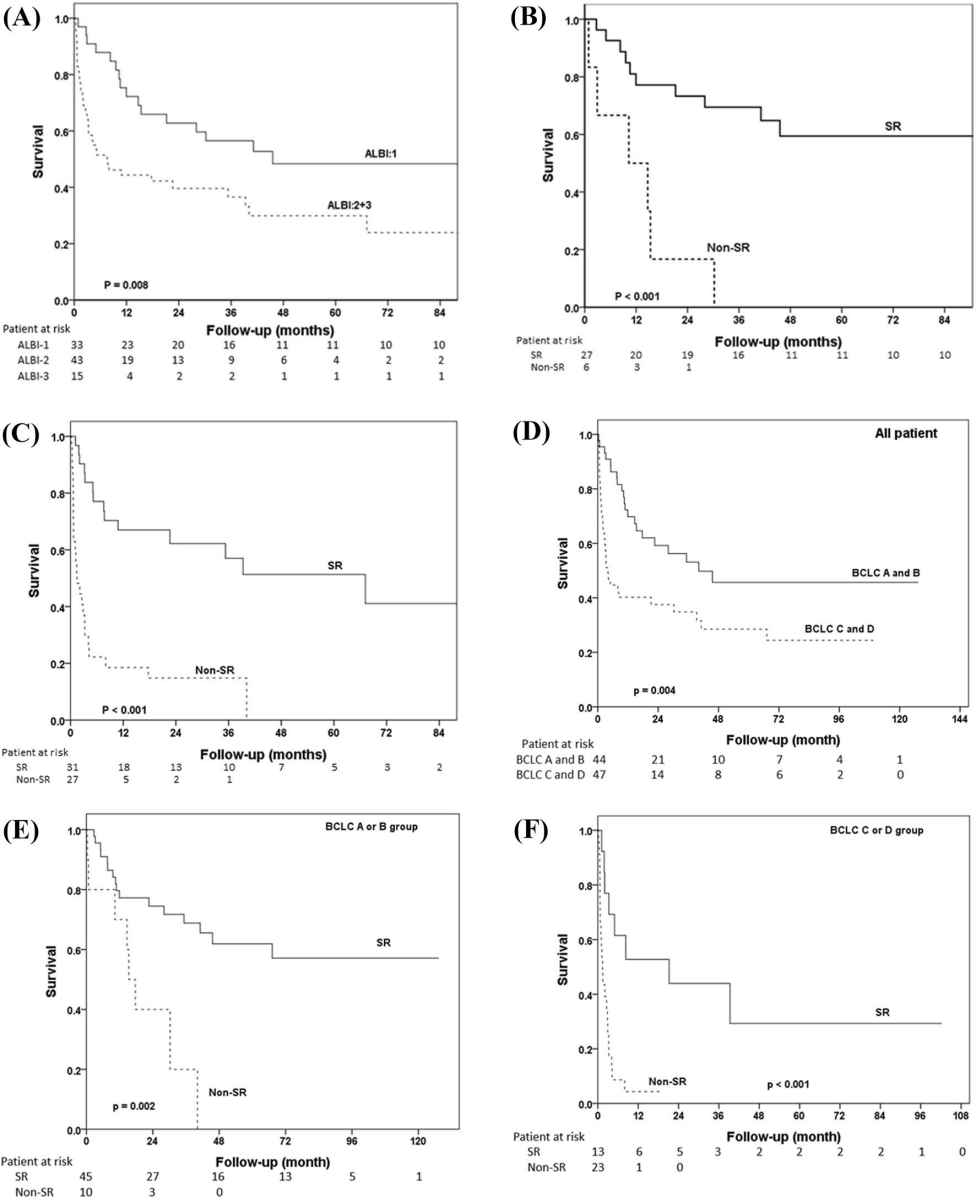
Comparison of OS rates between SR and non-SR groups stratified by ALBI grade and BCLC stages (**A**) Comparison of OS rates between patients with ALBI grade 1 and those with grade 2 or 3. (**B**) Comparison of OS rates between SR and non-SR groups in patients with ALBI grade 1. (**C**) Comparison of OS rates between SR and non-SR groups in patients with ALBI grade 2 or 3. (**D**) Comparison of OS rates between patients with BCLC stage A or B and those with BCLC stage C or D. (**E**) Comparison of OS rates between SR and non-SR groups in patients with BCLC stage A or B. (**F**) Comparison of OS rates between SR and non-SR groups in patients with BCLC stage C or D.

Next, we stratified the analysis by according to the ALBI grade. As shown in Fig. 3A, patients who had ALBI grade 1 had significantly longer OS than those with ALBI grade 2 or 3. Moreover, patients in the SR group had better prognoses than those in the non-SR group among both patients with ALBI grade 1 (Fig. 3B) and those with ALBI grade 2 or 3 (Fig. 3C).

When stratified the analysis by the BCLC stages, it showed that patients who had BCLC stage A or B had a higher OS rate than those with BCLC stage C or D in all patients (Fig. 3D). Besides, patients in the SR group had better prognoses than those in the non-SR group among both patients with BCLC stage A or B (Fig. 3E) and those with BCLC stage C or D (Fig. 3F).

### Multivariate analysis of the factors predictive of OS

As the ALBI scores were calculated using serum albumin and bilirubin levels, we applied two multivariate analysis models^9^. In model I, the ALBI grade was used, but serum albumin and bilirubin levels were not. In model II, we used serum albumin and bilirubin levels, but not the ALBI grade.

As shown in Table 2, model I revealed that the non-SR group (hazard ratio HR: 6.649, 95% CI 3.581–12.344, *p* < 0.001), serum α-fetoprotein (AFP) ≥ 100 mg/mL (HR 2.979, 95% CI 1.587–5.595, *p* = 0.001), hepatitis B surface (HBsAg) positivity (HR 0.368, 95% CI 0.200–0.678, *p* = 0.001), and ALBI grade 2 or 3 (HR: 2.013, 95% CI 1.091–3.711, *p* = 0.025) were the independent factors for predicting the OS for patients with ruptured HCC. Model II showed that the non-SR group (HR: 6.273, 95% CI 3.099–12.698, *p* < 0.001), serum AFP ≥ 100 mg/mL (HR: 3.083, 95% CI 1.412–6.729, *p* = 0.005), HBsAg positivity (HR: 0.411, 95% CI 0.198–0.850, *p* = 0.016), serum albumin levels ≤ 3.5 mg/dL (HR: 2.865, 95% CI 1.480–5.747, *p* = 0.004), and alkaline phosphatase (Alk-P) levels ≥ 100 U/L (HR: 1.956, 95% CI 1.006–3.804, *p* = 0.048) were the factors predictive of OS.

**Table 2:** Factors associated with poor OS in HCC patients with tumor rupture in univariate and model I multivariate analysis.

Variable	N (%)	Univariate analysis		Multivariate analysis	
		HR (95% CI)	*p*	HR (95% CI)	*p*
Non-SR/SR	33/58 (36.3/63.7)	6.173 (3.436–11.09)	< 0.001	6.649 (3.581–12.344)	< 0.001
Age (y/o) > 65/≤ 65	39/52 (42.9/57.1)	1.415 (0.824– 2.428)	0.208		
Gender M/F	71/20 (78.0/22.0)	0.858 (0.459–1.602)	0.630		
BMI (kg/m^2^) < 24/≥ 24	40/32 (44.0/35.2)	0.983 (0.555–1.825)	0.983		
AFP (ng/mL) ≥ 100/< 100	53/38 (58.2/41.8)	1.691 (0.961–2.973)	0.068	2.979 (1.587–5.595)	0.001
Size (cm) > 10/≤ 10	32/59 (35.2/64.8)	1.994 (1.161–3.426)	0.012		
HBsAg Y/N	42/48 (46.2/52.7)	0.570 (0.328–0.992)	0.047	0.368 (0.200–0.678)	0.001
Anti-HCV Y/N	16/73 (17.6/80.2)	1.934 (1.032–3.636)	0.040		
ALBI 2/3 & 1	58/33 (63.7/36.3)	2.179 (1.209–3.930)	0.010	2.013 (1.091–3.711)	0.025
MELD > 11/≤ 11	34/57 (37.4/62.6)	1.579 (0.919–2.711)	0.404		
Albumin (mg/dL) ≤ 3.5/> 3.5	42/47 (46.2/51.6)	2.652 (1.517–4.630)	0.001		
Platelet (/mm^3^) ≤ 150,000/> 150,000	26/65 (28.6/71.4)	1.270 (0.715–2.257)	0.415		
PTINR ≥ 1.15/ < 1.15	30/60 (33.0/65.9)	1.899 (1.094–3.296)	0.023		
Bilirubin (mg/dL) ≥ 1.2/< 1.2	29/62 (31.9/68.1)	1.817 (1.043–3.165)	0.035		
Hgb (mg/dL) ≤ 11/> 11	43/48 (47.3/52.7)	2.079 (1.212–3.571)	0.008		
BUN (mg/dL) ≥ 20/ < 20	29/58 (31.9/63.7)	2.087 (1.206–3.612)	0.009		
Creatinine (mg/dL) ≥ 1.0/ < 1.0	45/46 (49.5/50.5)	1.315 (0.770–2.249)	0.315		
ALT (U/L) ≥ 40/ < 40	36/55 (39.6/60.4)	1.738 (1.015/2.976)	0.044		
ALKP (U/L) ≥ 100/ < 100	33/40 (36.3/44.0)	1.973 (1.074–3.624)	0.028		
BCLC stage (C + D/A + B)	55/36 (60.4/39.6)	4.627 (2.651–8.077)	< 0.001		

*CI* confidence interval, *SR* surgical resection, *BMI* body mass index, *AFP* α-fetoprotein, *HBsAg* hepatitis B surface antigen, *HCV* hepatitis C virus, *MELD* model for end-stage liver disease, *ALBI* albumin-bilirubin, *PT INR* prothrombin time/international normalized ratio, *HgB* hemoglobulin, *ALT* alanine aminotransferase, *ALKP* alkaline phosphatase, *BCLC* Barcelona Clinic Liver Cancer.

Model II multivariate analysis showed that non-SR group (HR: 6.273, 95% CI: 3.099–12.698,* p* < 0.001), serum AFP ≥ 100 mg/mL (HR:3.083, 95% CI 1.412–6.729, *p* = 0.005), HBsAg positivity (HR:0.411, 95% CI 0.198–0.850, *p* = 0.016), serum albumin levels ≤ 3.5 mg/dL (HR 2.865, 95% CI 1.480–5.747, *p* = 0.004), and Alk-P levels ≥ 100 U/L (HR:1.956, 95% CI 1.006–3.804, *p* = 0.048) were the factors correlated with OS.

### Outcomes of HCC patients with tumor rupture in the SR group

Among the 58 patients in the SR group, 24 patients died during a median follow-up period of 34.1 (IQR 9.3–68.2) months. A multivariate analysis showed that serum AFP ≥ 100 (HR: 3.103, 95% CI 1.029–9.346, *p* = 0.044) and Alk-P ≥ 100 U/L (HR: 2.638, 95% CI 1.029–6.536, *p* = 0.036) were the independent factors associated with poor OS for patients with HCC rupture after SR (Supplementary Table S3). Sequential combination therapy of TACE and SR did not have survival benefit compared to SR alone (HR: 0.904, 95% CI 0.386–2.114, *p* = 0.816).

Furthermore, 28 patients had tumor recurrence after SR with a median recurrence-free survival (RFS) of 8.69 (IQR 4.23–36.59) months. The patterns of recurrence were intra-hepatic metastasis alone in 15 patients (53.6%), extra-hepatic recurrence alone in 7 patients (25.0%), and both intra- and extra-hepatic metastasis in 6 patients (21.4%), respectively. The number of sequential treatments after recurrence were re-resection in 8 patients, TACE in 5 patients, tyrosine kinase inhibitors (TKIs) in 5 patients, radiofrequency ablation in 3 patients, TACE with TKI combination therapy in 3 patients, immune checkpoint inhibitors (ICIs) in 2 patients, and BSC in 2 patients, respectively.

The cumulative RFS rates at 1 year, 2 years, and 3 years were 41.6%, 32.8%, and 24.1%, respectively. There was no statistically significant difference in RFS between those who received SR alone and those who received SR plus TACE (*p* = 0.828) (Supplementary Figure S1). Serum Alk-P level ≥ 100 U/L (HR: 2.370, 95% CI 1.170–4.808, *p* = 0.017), presence of macrovascular invasion (HR: 2.551, 95% CI 1.232–5.291, *p* = 0.012), and Ishak modified histologic activity index ≥ 3 (HR: 2.506, 95% CI 1.172–5.348, *p* = 0.018) were the factors associated with poor RFS for HCC patients with tumor rupture after SR (Supplementary Table S4).

## Discussion

There were several major findings in this study. First, we examined the real-world prognosis of HCC patients who experienced a tumor-rupture event in Taiwan, which showed that the 5 years OS rate was 36.7% in this clinical setting. Second, we aimed to figure out that the treatment modality that achieved better prognoses in patients with ruptured HCC. In our cohort, patients who underwent SR had an acceptable long-term outcome with a 5 years OS rate of 55.1%. In contrast, patients who received non-surgical treatment, such as TACE or BSC, had a median OS of 2.6 months. Obviously, SR was the strongest factor in determining the OS in rupture HCC patients, irrespective of the BCLC stages and the ALBI grades. Other risk factors included serum AFP level and liver functional reserve, which also had influences in OS. Third, we found that whether patients received TACE or not did not impact the survival in both the SR group and the non-SR group.

The incidence rate of ruptured HCC is reported as less than 3% in Western countries and as around 2.3–26% in all HCC cases in Asia^10^. In the recent decades, the incidence of ruptured HCC has decreased, which may be attributed to the successful implantation of surveillance programs for patients who have a high risk of developing HCC. Hence, more and more patients are diagnosed with HCC at an early stage^11^. However, HCC with tumor rupture is still one of the most fatal complications of HCC, with in-hospital or 30 days mortality rates as high as 30–70%^12^. Another nationwide survey from Japan showed that the 5 years OS rates were 13.3% and 45.8% in ruptured HCC patients and non-ruptured HCC patients, respectively^6^. In our cohort, the 5 years OS rate was 36.7% for patients with ruptured HCC, which was lower than those with non-ruptured HCC and reflected the poor prognosis of this population^13,14^.

In our cohort, 58 (63.74%) patients with HCC tumor rupture underwent SR with or without TACE in sequential combination therapy. Moreover, patients who underwent surgical intervention had better long-term survival than those with other treatments. This finding is consistent with other studies^10,15,16^. Of note, the 5 years OS rate was 55.1% in the SR group. Moreover, the long-term outcomes were similar between patients with and without TACE in the SR group. However, the median OS was only 2.6 months, and no one survived for more than 4 years when patients underwent non-SR treatment such as TACE or BSC. This suggests that patients with ruptured HCC should be considered to undergo SR if they have no contraindication, regardless of what therapies they received at the beginning of rupture event.

Since ruptured HCC is life-threatening emergency situation, TACE is regarded as the best method to employ for hemostasis, especially in unstable hemodynamic conditions^17,18^. However, although TACE could control the bleeding event immediately in our study, it did not produce a statistically significant difference in OS compared to BSC. The key factor for better survival outcome is surgical intervention, but it has limitations related to the tumor size, location, and preserved liver function. In some previous studies, emergency liver resection achieved good early and long-term results compared to TACE therapy^15,19^. Compared to other studies, the OS rates of patients in the SR group were better in our cohort. This may be attributed to the restriction of treatment-naïve HCC cases in our study and the rapid advances of novel systemic therapies in the last 5–10 years.

However, the prognosis in the TACE group was similar to that in other studies in that most patients died within half a year after TACE treatment without surgical intervention^20^. Since surgical treatment is important for these naïve HCC patients who experienced a rupture event, some investigators have recommended that one-stage hepatectomy be performed on patients with HCC in BCLC stages A and B^14^. Nevertheless, our study showed that one-stage SR and sequential combination therapy of TACE and SR had similar long-term outcomes in terms of both OS and RFS for patients with ruptured HCC. Further prospective studies are warranted to clarify this issue.

In this study, HBV and HCV infections were the major causes of HCC, which was consistent with previous reports^13,21–23^. However, there is still controversy about whether viral etiology plays an important role in determining the outcomes of patients with HCC^22–25^. This might be due to the discrepancy of liver functional reserve and tumor factors between HBV-related and HCV-related HCC patients. Previous studies show that patients with HBV-related HCC had more aggressive tumor phenotypes, but they had less liver cirrhosis and better liver functional reserve compared to those with HCV-related HCC^23,26^. In our cohort, patients with HBV-related HCC had better OS than those who did not. As all of the patients with advanced-stage HCC had tumor rupture at the time of diagnosis, liver functional reserve might be more important for determining the prognoses of patients.

Zhang and colleagues conducted a retrospective study which enrolled 101 patients with ruptured HCC^27^. In this study, the median OS were 5 days, 30 days, and 810 days, for patients who underwent conservative treatment, TACE, and SR, respectively. Although the 30 days mortality rate was 7.3% for patients who underwent SR, the long term post-operational outcomes of patients with ruptured HCC were similar to non-ruptured HCC patients. It indicated that SR could provide an acceptable long-term outcome for selected patients with ruptured HCC, which was consistent to our findings. However, this study did not compare the outcomes between patients who underwent SR alone and those with TACE and SR sequential combination therapy.

There were some limitations in this study. First, it was a cohort study that enrolled ruptured HCC patients at a single institution. Potential selection bias and missing data might exist due to the retrospective study design. Especially in patients who had better condition to afford SR might lead to the differences in results. Although we had performed multivariate analysis and subgroup analysis, including ALBI grades and BCLC stages, to reduce the impact of confounding factors on the comparison of prognoses between patients in the SR group and those in the non-SR group. These results must be interpreted with caution. Second, the major study populations were patients with viral hepatitis-related HCC from Asia. Further studies recruiting ruptured HCC patients with different ethnicities and non-viral hepatitis etiology are warranted to validate our study findings. Third, with the recent advances in the systemic therapy, more patients now receive TKI or ICI for advanced stage HCC, or as an adjuvant therapy after curative therapy or TACE. In our cohort, 12 patients in SR group and 5 patients in the non-SR group underwent systemic therapy after tumor progression (Supplementary S5). Nevertheless, due to the indication and regimens of systemic therapy were quite diverse in our cohort, we could not assess the impact of systemic therapy on the outcomes of patients with ruptured HCC. Further prospective studies are warranted to elucidate this issue. Fourth, the median follow-up time was relatively short in this study. In our cohort, the median follow-up is 34.1 (IQR 9.25–68.16) months in SR group, and 2.6 (IQR 0.68–12.50) months in non-SR group, respectively. Because more than 75% patients in non-SR group and 25% patients in SR group were dead within one year after diagnosis. Thus, the median level was fixed (13.1 months) even though some patients can be long-term survival more than 5 years in follow-up. It might reflect the poor outcomes of patients with ruptured HCC.

In conclusion, for patients with HCC and tumor rupture at the time of diagnosis, SR could provide better prognoses than non-surgery treatment modalities. Moreover, a sequential combination of TACE and SR had similar clinical outcomes in terms of both OS and RFS when compared to SR alone.

## Methods

### Patients

This study retrospectively reviewed 99 patients who were diagnosed with treatment-naïve HCC and tumor rupture at Taipei Veterans General Hospital from January 2008 to October 2020. Among them, 8 patients were excluded because tumor rupture occurred after treatment of HCC (Fig. 1). Hence, the remaining 91 patients were enrolled for the final analysis. The diagnosis of HCC was established according to the criteria of the American Association for the Study of Liver Disease (AASLD)^28^.

For each patient with newly diagnosed HCC at our hospital, potential treatment modalities were discussed in a weekly multidisciplinary HCC panel meeting attended by hepatologists, oncologists, surgeons, radiologists, pathologists, onco-radiologists, and nursing personnel^29–31^. The therapeutic decision was shared between the patient and the physician after discussing the risks, benefits, complications, efficacies of the potential treatments, and the recommendations from the multidisciplinary expert meeting.

Considering HCC tumor rupture is an emergent situation, the first treatment modality was decided by the patients and the emergency physicians, hepatologists, radiologists, and surgeons as soon as possible. If patients could overcome the emergent situation and get stabilized, then the weekly multidisciplinary HCC panel meeting would discuss the further treatment plan. The criteria of resectable HCC were as follows: (1) Child’s grade of liver function of A or B; (2) tumor involving no more than two Healey’s segments and without main portal vein trunk involvement; (3) absence of other major diseases that might complicate the surgery; and (4) absence of extra-hepatic tumor dissemination.

The baseline demographic characteristics, tumor stages, treatments, and outcomes of HCC patients were recorded in the HCC registration system and were updated every 3 months. The study was performed in accordance with the Declaration of Helsinki and current ethical guidelines. It was also approved by the Institutional Review Board (IRB) of Taipei Veterans General Hospital, Taiwan (VGHIRB No. 2021-05-008AC). As this study was a retrospective cohort study, the IRB of the Taipei Veterans General Hospital waived the requirement for informed consent. Patient information was de-identified before the initiation of this study.

### Statistical analysis

The primary endpoint of this study was OS. All patients were followed up until either their final hospital visit, death, or October 31, 2020. Fisher’s exact test or a chi-squared test with Yates’ correction were used to compare categorical variables when appropriate, and the Mann–Whitney *U*-test was used to compare continuous variables. Kaplan–Meier survival analysis was used to estimate OS and RFS after therapy. A Cox proportional hazards model was applied to determine the factors associated with OS. The variables with statistical significance (*p* < 0.05) or approximate significance (*p* < 0.1) in the univariate analysis were subjected to a multivariate analysis using a backward stepwise logistic regression model. All statistical analyses were performed using IBM SPSS Statistics for Windows, Version 21.0 (IBM Corp., Armonk, NY, USA).

## Supplementary Information

Supplementary Information.

## Data Availability

The datasets used and/or analysed during the current study available from the corresponding author on reasonable request.
