# Supplementary material for: Surgical resection could provide better outcomes for patients with hepatocellular carcinoma and tumor rupture
Source: Sci Rep. 2022 May 18;12:8343. doi: 10.1038/s41598-022-12350-x (PMC9117281; doi:10.1038/s41598-022-12350-x)
Supplement: Supplementary file 1 — Supplementary Information. [file 41598_2022_12350_MOESM1_ESM.docx]

**SUPPLEMENTARY INFORMATION**

**Title of manuscript: Surgical resection could provide better outcomes for patients with hepatocellular carcinoma and tumor rupture**

**Author list:**

Chun-Yang Lee, Gar-Yang Chau, Cheng-Yi Wei, Yee Chao, Yi-Hsiang Huang,

Teh-Ia Huo, Ming-Chih Hou, Yu-Hui Su, Jaw-Ching Wu, Chien-Wei Su

**Supplementary Table S1. Baselines demographics of** **SR alone and SR plus TACE**

|  | **SR alone** | **SR plus TACE** | **p** |
| --- | --- | --- | --- |
|  | **(n = 38)** | **(n = 20)** |  |
| **Age (years)** | 62.5, 52.0−76.2 | 60.5, 43.5−67.0 | 0.249 |
| **Gender (M/F) (%)** | 29/9 (76.3/23.7) | 16/4 (80.0/20.0) | 0.754 |
| **BMI (kg/m^2^)** | 23.6, 21.0−26.9 | 23.4, 20.4−25.8 | 0.826 |
| **AFP (ng/ml)** | 1160.9, 21.7−45599 | 287.7, 12.5−3501.3 | 0.598 |
| **Tumor size (cm)** | 8.9, 6.1−11.1 | 6.2, 4.6−10.0 | 0.050 |
| **HBsAg (+/−) (%)** | 20/18 (52.6/47.4) | 11/9 (55.0/45.0) | 0.866 |
| **Anti-HCV (+/−)(%)** | 5/32 (13.5/86.5) | 3/17 (15.0/85.0) | 0.880 |
| **Albumin(mg/dL)** | 4.0, 3.5−4.1 | 3.5, 3.2−4.0 | 0.206 |
| **Bilirubin (U/L)** | 0.72, 0.51−0.98 | 0.99, 0.62−1.68 | 0.067 |
| **Platelet (/mm^3^)** | 222000,  169500−254750 | 212500,  136000−276750 | 0.773 |
| **PT INR** | 1.08, 1.02−1.13 | 1.11, 1.05−1.19 | 0.209 |
| **Hgb (mg/dL)** | 12.8, 11.3−14.5 | 11.1, 9.5−12.4 | 0.016 |
| **BUN (mg/dL)** | 14, 12.0−18.2 | 16.0, 11.0−22.0 | 0.185 |
| **Creatinine (mg/dL)** | 0.97, 0.80−1.21 | 0.92, 0.81−1.16 | 0.287 |
| **ALT (U/L)** | 29.5, 23.7−51.2 | 32.0, 28.0−-85.2 | 0.118 |
| **MELD** | 7.59, 6.89−8.80 | 8.16, 7.34−17.3 | 0.592 |
| **ALBI** | -2.62, −2.84~−2.19 | -2.21, −2.67~−1.87 | 0.039 |
| **ALBI (1/2/3) (%)** | 21/15/2 (55.3/39.5/5.3) | 6/13/1(30.0/65.0/5.0) | 0.128 |
| **Child-Pugh class (A/B/C) (%)** | 36/2/0 (94.7/5.3/0) | 15/5/0 (75.0/25.0/0) | 0.028 |
| **BCLC stage**  **(A/B/C/D) (%)** | 6/25/7/0 (15.8/65.8/18.4/0) | 4/10/6/0 (20/50/30/0) | 0.677 |

Continuous variables are expressed as the median with 25th and 75th percentiles.

Abbreviations: SR: surgical resection; TACE: trans-arterial chemoembolization; BMI: body mass index; AFP, α-fetoprotein; HBsAg, hepatitis B surface antigen; HCV, hepatitis C virus; MELD, model for end-stage liver disease; ALBI, albumin-bilirubin; PT INR, prothrombin time/international normalized ratio; HgB, hemoglobulin; ALT, alanine aminotransferase; BCLC, Barcelona Clinic Liver Cancer

**Supplementary Table S2. Baselines demographics of TACE alone and BSC**

|  | **TACE alone** | **BSC** | **p** |
| --- | --- | --- | --- |
|  | **(n = 28)** | **(n = 5)** |  |
| **Age (years)** | 62.5, 52.0−76.2 | 68.0, 51.5−86.0 | 0.690 |
| **Gender (M/F) (%)** | 22/6 (78.6/21.4) | 4/1 (80.0/20.0) | 0.945 |
| **BMI (kg/m^2^)** | 23.7, 21.7−27.4 | 23.9, 19.9−26.0 | 0.617 |
| **AFP (ng/ml)** | 441.0, 11.4−16542.5 | 623.8, 21.8−174178.9 | 0.874 |
| **Tumor size (cm)** | 8.1, 5.0−13.7 | 12.0, 10.5−15.9 | 0.317 |
| **HBsAg (+/−) (%)** | 10/17 (37.0/63.0) | 1/4 (20.0/80.0) | 0.477 |
| **Anti-HCV (+/−)(%)** | 5/22 (18.5/81.5) | 3/2 (60.0/40.0) | 0.051 |
| **Albumin(mg/dL)** | 2.9, 2.7−3.4 | 3.9, 2.75−4.32 | 0.050 |
| **Bilirubin (U/L)** | 1.09, 0.64−1.72 | 0.60, 0.50−1.57 | 0.390 |
| **Platelet (/mm^3^)** | 167000,  91500−265250 | 228000,  153000−325000 | 0.240 |
| **PT INR** | 1.20, 1.08−1.33 | 1.06, 1.00−1.16 | 0.149 |
| **Hgb (mg/dL)** | 9.1, 7.4−10.5 | 12.8, 8.75−13.4 | 0.058 |
| **BUN (mg/dL)** | 22, 12.7−32.0 | 18.5, 11.5−28.5 | 0.538 |
| **Creatinine (mg/dL)** | 1.14, 0.80−1.76 | 0.98, 0.65−5.25 | 0.346 |
| **ALT (U/L)** | 36.5, 25.2−95.2 | 75.0, 33.5−-86.5 | 0.587 |
| **MELD** | 11.96, 9.54−13.79 | 10.71, 9.24−15.5 | 0.829 |
| **ALBI** | -1.67, -2.05~−1.15 | -2.65, −2.95~−1.41 | 0.782 |
| **ALBI (1/2/3) (%)** | 3/15/10 (10.7/53.6/35.7) | 3/0/2 (60.0/0/40.0) | 0.207 |
| **Child-Pugh class (A/B/C) (%)** | 10/13/3 (38.5/50/11.5) | 3/2/0 (75.0/25.0/0) | 0.307 |
| **BCLC stage**  **(A/B/C/D) (%)** | 1/7/14/6  (3.6/25/50/21.4) | 0/2/2/1  (0/40/40/20) | 0.811 |

Continuous variables are expressed as the median with 25th and 75th percentiles.

Abbreviations: SR: surgical resection; TACE: trans-arterial chemoembolization; BMI: body mass index; AFP, α-fetoprotein; HBsAg, hepatitis B surface antigen; HCV, hepatitis C virus; MELD, model for end-stage liver disease; ALBI, albumin-bilirubin; PT INR, prothrombin time/international normalized ratio; HgB, hemoglobulin; ALT, alanine aminotransferase; BCLC, Barcelona Clinic Liver Cancer

**Supplementary Table S3. The univariate and multivariate analyses for the factors predictive of poor OS of patients in the SR group**

|  |  | **Univariate analysis** | | **Multivariate analysis** | |
| --- | --- | --- | --- | --- | --- |
| **Variable** | **N (%)** | **HR (95% CI)** | **p** | **HR (95% CI)** | **p** |
| **Age (y/o) ≤65/>65** | 36/22 (62.1/37.9) | 1.418 (0.622−3.229) | 0.406 |  |  |
| **Gender M/F** | 45/13 (77.6/22.4) | 1.160 (0.460−2.926) | 0.754 |  |  |
| **BMI (kg/m^2^) <24/≥24** | 27/19 (58.7/41.3) | 0.920 (0.375−2.257) | 0.855 |  |  |
| **AFP (ng/mL) ≥100/<100** | 34/24 (58.6/41.4) | 3.092 (1.215−7.869) | 0.018 | 3.106 (1.029−9.346) | 0.044 |
| **Size (cm) >10/≤10** | 16/42 (27.6/72.4) | 1.913 (0.835−4.382) | 0.125 |  |  |
| **HBsAg Y/N** | 31 (53.4) | 0.758 (0.502−2.575) | 0.758 |  |  |
| **Anti-HCV Y/N** | 8 (14.0) | 1.652 (0.612−4.464) | 0.321 |  |  |
| **ALBI 2/3 & 1** | 31/27 (53.4/46.6) | 1.642 (0.725−3.715) | 0.234 |  |  |
| **MELD >11/≤11** | 13/45 (22.4/77.6) | 2.724 (0.811−9.174) | 0.105 |  |  |
| **Albumin (mg/dL) ≤3.5/>3.5** | 19/39 (32.8/67.2) | 1.754 (0.778−3.952) | 0.175 |  |  |
| **Platelet (/mm^3^) ≤150000/>150000** | 12/46 (20.7/79.3) | 1.20 (0.339−2.404) | 0.828 |  |  |
| **PT INR ≥1.15/<1.15** | 13/44 (22.8/77.2) | 1.522 (0.603−3.844) | 0.374 |  |  |
| **Bilirubin (mg/dL) ≥1.2/<1.2** | 15/43 (74.1/25.9) | 1.215 (0.481−3.067) | 0.680 |  |  |
| **Hgb (mg/dL) ≤11/>11** | 18/40 (31.0/69.0) | 1.035 (0.429−2.500) | 0.939 |  |  |
| **BUN (mg/dL) ≥20/<20** | 13/44 (22.8/77.2) | 1.657 (0.708−3.875) | 0.244 |  |  |
| **Creatinine (mg/dL) ≥1.0/<1.0** | 26/32 (44.8/55.2) | 1.283 (0.576−2.859) | 0.542 |  |  |
| **ALT (U/L) ≥40/<40** | 20/38 (34.5/65.5) | 1.610 (0.714/3.629) | 0.251 |  |  |
| **ALKP (U/L) ≥100/<100** | 21/27 (43.8/56.3) | 2.926 (1.181-7.246) | 0.020 | 2.638 (1.029−6.536) | 0.036 |
| **TACE Y/N** | 38/20 (65.5/34.5) | 0.904 (0.386-2.114) | 0.816 |  |  |
| **BCLC C or D/ A or B** | 13/45 (22.4/77.6) | 2.732 (1.162-6.410) | 0.021 |  |  |
| **Surgery type-Lobectomy Y/N** | 20/38 (34.5/65.5) | 1.416 (0.562-3.570) | 0.461 |  |  |
| **Macro-vascular invasion Y/N** | 16/42 (27.6/72.4) | 2.618 (1.157-5.952) | 0.021 |  |  |
| **Micro-vascular invasion Y/N** | 52/6 (89.7/10.3) | 0.306 (0.041-2.264) | 0.246 |  |  |
| **Extra-hepatic capsule extension Y/N** | 53/5 (91.4/8.6) | 1.550 (0.357-6.725) | 0.558 |  |  |
| **Tumor cell differentiate ≥ 3/<3 (Edmondson and Steiner)** | 18/40 (31.0/69.0) | 0.480 (0.207-1.117) | 0.089 |  |  |
| **Ishak Modified HAI grading ≥ 3/<3** | 19/39 (32.8/67.2) | 0.448 (0.193-1.038) | 0.061 |  |  |
| **Ishak fibrosis score ≥ 4/<4** | 24/34 (41.4/58.6) | 0.651 (0.292-1.449) | 0.293 |  |  |

Abbreviations: CI, confidence interval; SR, surgical resection; BMI, body mass index; AFP, α-fetoprotein; HBsAg, hepatitis B surface antigen; HCV, hepatitis C virus; MELD, model for end-stage liver disease; ALBI, albumin-bilirubin; PT INR, prothrombin time/international normalized ratio; HgB, hemoglobulin; ALT, alanine aminotransferase; ALKP, alkaline phosphatase; BCLC, Barcelona Clinic Liver Cancer; HAI, histologic activity index.

**Supplementary Table S4. The univariate analysis for the factors associated with poor RFS of patients in the SR group**

|  |  | **Univariate analysis** | | **Multivariate analysis** | |
| --- | --- | --- | --- | --- | --- |
| **Variable** | **N (%)** | **HR (95% CI)** | **p** | **HR (95% CI)** | **P** |
| **Age (y/o) >65/≤65** | 36/22 (62.1/37.9) | 1.235(0.655-2.327) | 0.514 |  |  |
| **Gender M/F** | 45/13 (77.6/22.4) | 0.827(0.404-1.689) | 0.603 |  |  |
| **BMI (kg/m^2^) <24/≥24** | 27/19 (58.7/41.3) | 0.827(0.414-1.652) | 0.592 |  |  |
| **AFP (ng/mL) ≥100/<100** | 34/24 (58.6/41.4) | 1.406 (0.752−2.630) | 0.286 |  |  |
| **Size (cm) >10/≤10** | 16/42 (27.6/72.4) | 1.353 (0.688−2.659) | 0.381 |  |  |
| **HBsAg Y/N** | 31/27 (53.4/46.6) | 0.982 (0.525−1.837) | 0.954 |  |  |
| **Anti-HCV Y/N** | 8/49 (14.0/86.0) | 1.785 (0.807−3.952) | 0.152 |  |  |
| **ALBI 2/3 & 1** | 31/27 (53.4/46.6) | 1.179 (0.636−2.188) | 0.601 |  |  |
| **MELD >11/≤11** | 13/45 (22.4/77.6) | 1.293 (0.632−2.645) | 0.481 |  |  |
| **Albumin (mg/dL) ≤3.5/>3.5** | 19/39 (32.8/67.2) | 1.600 (0.842−3.039) | 0.151 |  |  |
| **Platelet (/mm^3^) ≤150000/>150000** | 12/46 (20.7/79.3) | 1.253 (0.575−2.728) | 0.570 |  |  |
| **PT INR ≥1.15/<1.15** | 13/44 (22.8/77.2) | 1.304 (0.621−2.737) | 0.484 |  |  |
| **Bilirubin (mg/dL) ≥1.2/<1.2** | 15/43 (74.1/25.9) | 1.590 (0.805−3.141) | 0.182 |  |  |
| **Hgb (mg/dL) ≤11/>11** | 18/40 (31.0/69.0) | 1.584 (0.830−3.021) | 0.162 |  |  |
| **BUN (mg/dL) ≥20/<20** | 13/44 (22.8/77.2) | 1.071 (1.412−2.188) | 0.850 |  |  |
| **Creatinine (mg/dL) ≥1.0/<1.0** | 26/32 (44.8/55.2) | 1.019 (0.550−1.891) | 0.951 |  |  |
| **ALT (U/L) ≥40/<40** | 20/38 (34.5/65.5) | 1.629 (0.870/3.052) | 0.127 |  |  |
| **ALKP (U/L) ≥100/<100** | 21/27 (43.8/56.3) | 2.710 (1.360-5.401) | 0.005 | 2.370 (1.170-4.808) | 0.017 |
| **TACE Y/N** | 38/20 (65.5/34.5) | 0.939 (0.492-1.792) | 0.848 |  |  |
| **BCLC C/ A or B** | 13/45 (22.4/77.6) | 0.277 (1.808-7.194) | <0.001 |  |  |
| **Surgery type-Lobectomy Y/N** | 20/38 (34.5/65.5) | 0.832 (0.436-1.589) | 0.577 |  |  |
| **Macro-vascular invasion Y/N** | 16/42 (27.6/72.4) | 2.865 (1.052-5.464) | 0.001 | 2.551 (1.232-5.291) | 0.012 |
| **Micro-vascular invasion Y/N** | 52/6 (89.7/10.3) | 7.300 (1.001-52.631) | 0.050 |  |  |
| **Extra-hepatic capsule extension Y/N** | 53/5 (91.4/8.6) | 2.326 (0.810-6.681) | 0.117 |  |  |
| **Tumor cell differentiate ≥ 3/<3 (Edmondson and Steiner)** | 18/40 (31.0/69.0) | 0.668 (0.343-1.300) | 0.235 |  |  |
| **Ishak Modified HAI grading ≥ 3/<3** | 19/39 (32.8/67.2) | 2.119 (1.092-4.115) | 0.032 | 2.506 (1.172-5.348) | 0.018 |
| **Ishak fibrosis score ≥ 4/<4** | 24/34 (41.4/58.6) | 0.825 (0.444-1.530) | 0.541 |  |  |

Abbreviations: CI, confidence interval; SR, surgical resection; BMI, body mass index; AFP, α-fetoprotein; HBsAg, hepatitis B surface antigen; HCV, hepatitis C virus; MELD, model for end-stage liver disease; ALBI, albumin-bilirubin; PT INR, prothrombin time/international normalized ratio; HgB, hemoglobulin; ALT, alanine aminotransferase; ALKP, alkaline phosphatase. TACE, transarterial chemoembolization, BCLC, Barcelona Clinic Liver Cancer; HAI, histologic activity index.

**Supplementary Table S5. Systemic therapy after tumor tumor progression**

| Number of patients | Systemic therapy | Number of patients | Systemic therapy |
| --- | --- | --- | --- |
| SR group  (n=12) | | Non-SR group  (n=5) | |
| 6 | Sorafenib | 2 | Lenvatinib, Nivolumab |
| 1 | Durvalumab with Tremelimumab | 1 | Sorafenib, Regorafenib |
| 1 | Pembrolizumab | 1 | Sorafenib |
| 1 | Everolimus | 1 | Dovitinib |
| 1 | Sorafenib, Regorafenib, Lenvatinib |  |  |
| 1 | Linifanib |  |  |
| 1 | Sorafenib, Nivolumab |  |  |

Abbreviations: SR, surgical resection


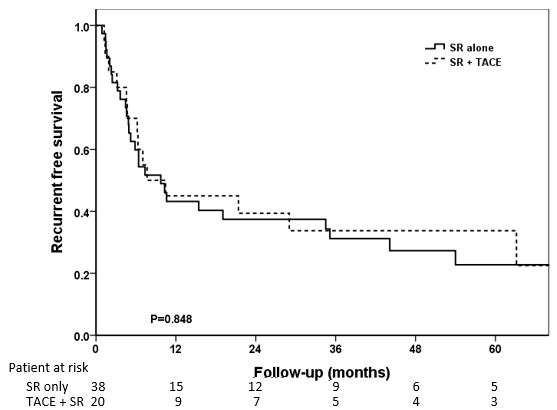


**Supplementary Figure S1.** Comparison of RFS between patients who underwent SR alone and those with a sequential combination therapy of TACE and SR.
